# 

**Published:** 1896-09

**Authors:** 


### Tri-State Medical Society.

The following has been issued:

You are invited, with your friends, to attend the Eighth Annual Meeting of the Tri-State Medical Society of Alabama, Georgia and Tennessee, to be held in Chattanooga, Tuesday, Wednesday and Thursday, October 13, 14 and 15, 1896.

DR. FRANK TRESTER SMITH, Secretary,
Chattanooga, Tenn.

DR. J. B. MURFREE, President, Murfreesboro, Tenn.

**Austin District Medical Society.**—The thirty-sixth quarterly meeting of the Austin District Medical Society will be held in Austin on Thursday, September 24th, 1896. An unusually interesting programme has been arranged, and the members of the regular profession are cordially invited to meet with us and participate in the discussions. We promise you a pleasant and profitable time.

S. E. HUDSON, Secretary.

